# Transcriptome Analysis Reveals the Mechanism of Natural Ovarian Ageing

**DOI:** 10.3389/fendo.2022.918212

**Published:** 2022-07-14

**Authors:** Lili Chen, Bo Ding, Liju Wu, Jialing Qiu, Qiong Li, Zheng Ye, Jinmei Yang

**Affiliations:** ^1^ Anhui University of Traditional Chinese Medicine Affiliated Chuzhou Hospital of Integrated Chinese and Western Medicine, Chuzhou, China; ^2^ Southeast University Affiliated Zhongda Hospital, Nanjing, China

**Keywords:** ovary, aging, mechanism, inflammation, reproduction

## Abstract

**Background:**

The decline in the quantity and quality of oocytes due to ovarian ageing in women is now a significant threat to reproductive health today as the concept of delayed fertility becomes widespread. However, the molecular mechanisms of natural ovarian ageing have not been fully elucidated.

**Method:**

Here, we used transcriptomic data from 180 normal ovarian tissues from GTEx V8 to analyze the expression profile of ovarian tissues from women with age segments of 20-29 (22 individuals), 30-39 (14 individuals), 40-49 (37 individuals), 50-59 (61 individuals), 60-69 (42 individuals), and 70-79 (4 individuals), respectively. XCELL was used to assess the infiltration score of 64 cell types of the ovary. WGCNA was used to characterize the co-expression network during the natural aging of the ovary. ClusterprofileR was used for functional enrichment analysis of co-expression modules. MsViper was used for master regulator analysis.

**Results:**

The infiltration score of endothelial cells and activated antigen-presenting cells during natural ovarian ageing increased significantly at ages 30-39, 40-49, and then decreased, whereas CD4+ Tcm increased with age. WGCNA identified six co-expression modules from ovarian tissue transcriptomic data species. The red module was significantly and positively correlated with senescence and CD4+ Tcm, and the turquoise module was significantly and positively correlated with Endothelial Cells. We further explored ovarian tissue for women aged 20-29 and 30-39 years. The GSEA results showed that the Chemokine signaling pathway was significantly activated in the 30-39-year-old group, while Oocyte meiosis was significantly inhibited. Finally, the results of msviper found that transcription factors such as KDM1A, PRDM5, ZNF726, PPARG, FOXJ2, and GLI2 were mainly activated in the 20-29 years group, while VAV1, RUNX3, ZC3H12D, MYCL, and IRF5 were mainly activated in the 30-39 years group and that these transcription factor activities were diagnostic of natural ovarian ageing (AUC: 0.65-0.71).

**Conclusion:**

Natural ageing of the ovary is significantly correlated with immune cell infiltration and activation of inflammation-related signaling pathways, with inflammation levels reaching a maximum during early ovarian ageing (30-39, 40-49) and then gradually decreasing after that. These studies provide a research basis for exploring the mechanisms of natural ovarian ageing.

## Background

With socio-economic development, delaying childbirth has become more accepted by most people. However, delaying childbirth can cause a unique and compelling problem for women. Women have a limited number of oocytes throughout their lives. The oocytes stored in the follicular pool constitute the ovarian reserve, and the quantity and quality of these cells are essential for the health status of the ovaries (1). The normal onset of fertility decline in women is around 32 years of age and around 51 years when the ovarian follicles fail, and fertility is wholly lost (2, 3). Premature ovarian failure can lead to amenorrhoea before the age of 40 and is associated with infertility (4). These studies have shown that ovarian ageing is an important cause of infertility in middle-aged women due to a decline in the number and quality of oocytes (5, 6). An elevated basal inflammatory state is one of the critical markers of ageing (7). The inflammatory microenvironment is associated with many ageing-related diseases (8), including arthritis (9), type II diabetes (10), and cardiovascular disease (11). It has been shown that local inflammation can disrupt ovarian function and oocyte development (12). Obesity-induced activation of ovarian inflammatory pathways may have devastating effects on follicle development and oocyte quality (13, 14). Although a large number of studies have shown an important relationship between inflammation and ovarian ageing (15), there are still no systematic studies that elucidate the course of ovarian ageing throughout the life cycle. In this study, we conducted a systematic study using transcriptomic data from 180 ovarian samples in the age range 20-79, focusing on the process of changes in immune cells and immune checkpoint-related genes in the gene expression profile of ovarian tissue during natural ageing. In addition, we conducted an in-depth analysis of ovarian transcriptome data in the 20-29 and 30-39 age groups and found that inflammation-associated signalling pathways and inflammation-associated transcription factors showed significant activation in the reproductive age group (30-39). These results reveal the relationship between the natural ageing process of the ovary and the ovarian immune microenvironment and provide a theoretical basis for the care of ageing ovaries and the treatment of female infertility.

## Materials and Methods

### Data Sources

**Figure 1:**
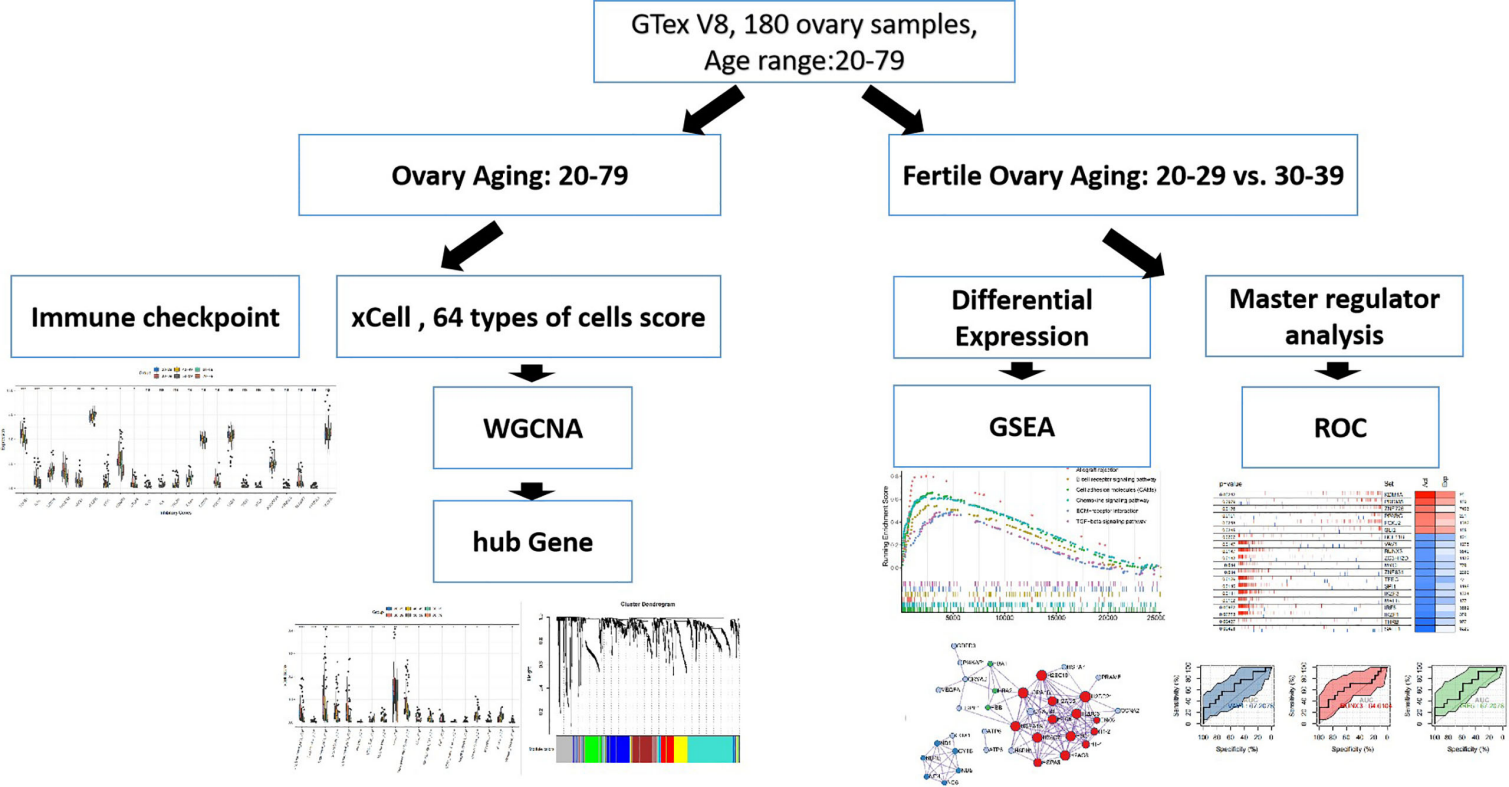
WorkFlow.

All samples involved in this study were obtained from ovarian tissue samples in the GTEx (16) V8 dataset. Clinical information for the samples is in Appendix1. The workflow is shown in **Figure 1**.

### Comparison of Immune Cell Scores and Immune Checkpoint Gene Expression

XCELL (17) was used to assess the infiltration score of 64 cell types and three composite scores (StromaScore, Immune Score, MicroenvironmentScore) in 180 ovarian samples. The gene sets for ImmuneScore and StromaScore were derived from the ESTIMATE algorithm (18). We scored these two gene sets using XCELL. microenvironmentScore is the sum of the immuneScore and StromaScore. During data processing, transcriptomic data were normalized using a logarithmic transformation (log2 (TPM+1). XCELL uses default parameters, and the results obtained from the assessment are stored in Appendix1. Immune checkpoint molecules were obtained from the Immunport database (https://www.immport.org/home). The mv_Endothelial_cell refers to mitral valve endothelial cell. The ly_Endothelial_cell refers to lymphatic endothelial cell. The aDC refer to activated Dendritic Cell.

### Weighted Gene Co-Expression Network Analysis

WGCNA (19) analyzed co-expressed module genes in ovarian samples. We calculated the Pearson correlation of each module with these scores based on age information scores, activated DC cell scores, endothelial cell scores, and CD4+ Tcm scores included in the analysis. WGCNA algorithm allows the construction of scale-free networks between genes based on their expression information and then clustering of the closest genes. We selected genes with standard deviation >1 as input and merged genes with module distance <0.25. The enrichGO function in clusterprofileR (20) was used to perform functional enrichment analysis on genes with hub Gene (screening criteria Module Membership >0.7) in the gene set (Appendix1).

### Differential Expression Analysis and Functional Enrichment Analysis

DESeq2 (21) was used to analyze differentially expressed genes in the 20-29 and the 30-39 age groups. Differential expression analysis was performed using Raw Count. Log2Foldchange>1, adjusted p-value <0.05 as differentially expressed gene screening criteria. ClusterprofileR was used to perform GSEA (22) (based on log2Foldchange sorting) on differentially expressed genes. We used the 186 gene set of MSIGDB C2 KEGG as a target for enrichment, with an adjusted p-value < 0.05 being considered significantly enriched. Metascape (23) was used to analyze the function, Protein-Protien-Interaction network, and coregulatory network of ageing-related genes.

### Master Regulator Analysis

Msviper (24) analyzed master regulators in groups 20-29 and 30-39. ARACNE-AP first assessed the regulatory network of transcription factors based on 36 samples (Appendx2). Then transcription factor activity was assessed for groups 20-29 and 30-39 using msviper. In addition, the viper was used to assess transcription factor activity for each sample, and logistic regression models were constructed based on transcription factor activity to find markers of transcription factor activity in ovarian senescence. The logistic regression model is used to construct the classifier, and the ROC curve is used to assess the predictive value of the model.

### Statistics

R 4.10 was used to perform statistical analyses. The R package ggpubr (25) was used for statistical plots and statistical tests. enrichplot was used to plot the results of GSEA. p<0.05 was considered statistically significant. *P < 0.05; ** P < 0.01; *** p<0.005; **** p<0.001; ns Not Significant.

## Results

### Patterns of Cellular Changes in the Ovarian Microenvironment During Ovarian Aging

**Figure 2:**
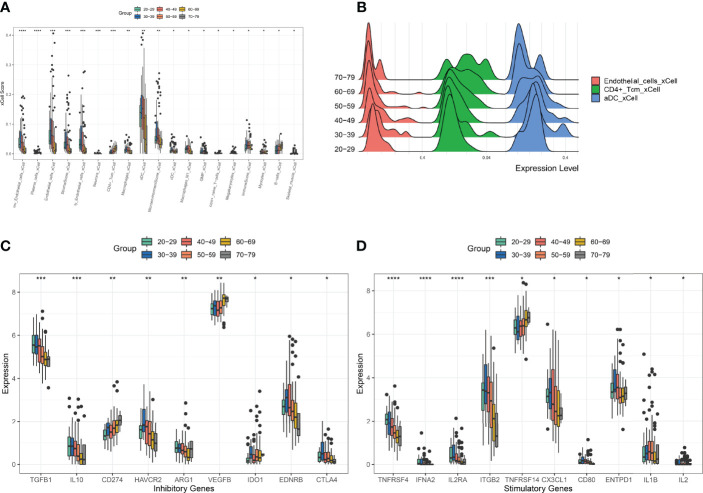
Comparison of XCELL scores of ovarian tissues. **(A)** Cell types with significant differences. **(B)** Distribution of Endotheial cells, CD4+ Tcm, aDC in the six age groups. **(C)** Comparison of immune-inhibitory checkpoint gene expression. **(D)** Comparison of gene expression in immune-stimulatory checkpoints. *p < 0.05, **p < 0.01, ***p < 0.005, ****p < 0.001.

To characterise changes in the ovarian microenvironment during natural ovarian ageing, xcell was used to assess the infiltration scores of 64 cell types in 180 ovarian tissue samples ranging in age from 20-79 years. The results showed mv Endothelial cells, plasma cells, Endothelial cells, Stroma, ly Endothelial cells, Neurons, CD4+ Tcm, Macrophages, aDC, MicroenvironmentScore, GMP, CD8+ naive T, Megakaryocytes, ImmuneScore, Myocytes, B cells, Skeletal muscle were significantly different in six subgroups (kruskal-wallis test, p<0.05, **Figures 2A, B**). Among these cells, we found that mv Endothelial cells, Endothelial cells, ly Endothelial cells, Macrophages, aDC reached their highest at 30-39 and then started to decrease progressively with age; CD4+ Tcm increased with age; B cells, on the other hand, do not change significantly at ages 20-49 and begin to rise significantly after ages 50-59. These details reflect that immune cells and endothelial cells are the cells that change most markedly during the natural ageing of the ovary. Elevated levels of basal inflammation are a typical feature of ageing. To further explore the relationship between immune cells and natural ovarian ageing, we compared the expression levels of immune checkpoint molecules across six age groups. Comparison of immunosuppressive checkpoint genes showed that TGFB1, IL10, HAVCR2, and EDNRB decreased progressively with age, whereas CD274, VEGFB increased significantly (**Figure 2C**). Comparison of immune checkpoint activation genes showed that the expression levels of TNFRSF4 and ITGB2 decreased with age, whereas TNFRSF14 increased progressively (**Figure 2D**). Having previously identified immune-related cells significantly increased between the ages of 30-39 years, we wanted to identify key immune checkpoint genes that drive immune activation. The immune activation genes TNFRSF4, IL2RA, and ITGB2 were highly variable throughout ovarian ageing, particularly in the 30-39 age range. Therefore, the use of these genes as targets may be important in delaying natural ovarian ageing. Similarly, the enhancement of the function of the immunosuppressive genes TGFB1, HAVCR2, EDNRB and CTLA4 is also relevant to the resistance of the ovary to natural ageing. These results suggest a unique pattern of activation and suppression of immune checkpoint-related genes during natural ovarian ageing. These immune checkpoints may serve as targets for drugs that inhibit the natural ageing of the ovary.

### Analysis of Weighted Gene Co-Expression Modules for Natural Aging of the Ovary

**Figure 3:**
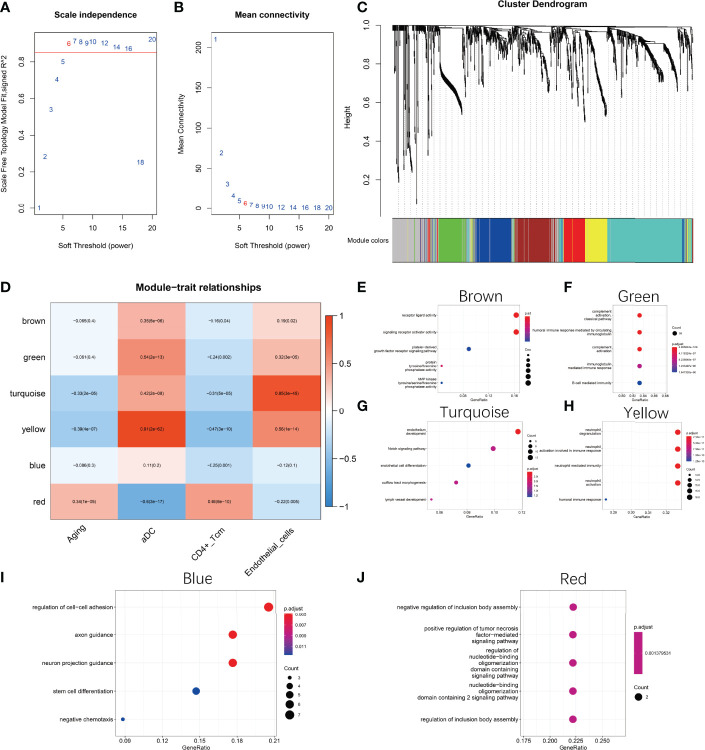
Weighted gene co-expression network analysis (WGCNA). **(A, B)** Selection of the best soft threshold. **(C)** Six co-expression modules were obtained for genes with fusion distance less than 0.25. Among them, the grey modules contained genes that could not be clustered. **(D)** Correlation of modules with phenotypes. **(E)** Functional enrichment analysis of Brown module hub genes **(F)** Functional enrichment analysis of Green module hub genes **(G)** Functional enrichment analysis of Turquoise module hub genes **(H)** Functional enrichment analysis of Yellow module hub genes **(I)** Functional enrichment analysis of Blue module hub genes **(J)** Functional enrichment analysis of Red Module hub genes functional enrichment analysis. The hub genes of each module are selected based on MM values greater than 0.7.

To explore the core regulatory modules during natural ovarian ageing, we performed WGCNA on 180 ovarian tissues. Genes with a standard deviation greater than 1.0 were used as input. Characteristics used for the clinical correlation analysis included age grading, aDC, CD4+ Tcm, and Endothelial cells score. By selecting a soft threshold of 6 and merging genes with a distance of less than 0.25, we ended up with 7 modules (Appendix1, **Figures 3A, B**). By selecting a soft threshold of 6 and merging genes with a distance of less than 0.25, we ended up with 7 modules (Appendix1, **Figures 3A, B**). The grey modules contain modules composed of genes that could not be clustered with other genes. We found that the red module was significantly positively correlated with Aging and CD4+ Tcm (r=0.34, p<0.001; r=0.45, p<0.001) and significantly negatively correlated with aDC (r=-0.6, p<0.001). This result illustrates that the red module is the most dominant regulatory module of ovarian aging. In addition, we found that the yellow and turquoise modules were significantly negatively correlated with senescence (yellow module:r=-0.39, p<0.001; turquoise module: r=-0.33, p<0.001), while significantly positively correlated with aDC (yellow module: r=0.91, p<0.001; turquoise module: r=0.42, p<0.001) and Endothelial cellss (yellow module: r=0.56, p<0.001; turquoise module: r=0.85, p<0.001) (**Figure 3D**). The results of the functional enrichment analysis indicate that the hub genes of the Brown module were mainly enriched in signalling pathways such as receptor ligand activity, signaling receptor activator activity, platelet-derived growth factor receptor signaling pathway, protein tyrosine/threonine phosphatase activity, MAP kinase tyrosine/serine/threoine phosphatase activity (**Figure 3E**). The hub genes of the Green module were mainly enriched in the complement activation classical pathway, humoral immune response mediated by circulating immunoglobulin, complement activation, immunoglobulin mediated immune response, B cell mediated immunity signaling pathways (**Figure 3F**). Turquoise modules were mainly enriched in signaling pathways such as endothelium development, Notch signaling pathway, endothelial cell differentiation, outflow tract morphogenesis, and lymph vessel development (**Figure 3G**). The hub genes of the Yellow module were enriched in signaling pathways such as neutrophil degranulation, neutrophil activation involved in immune response, neutrophil-mediated immunity, neutrophil activation, and humoral immune response (**Figure 3H**). The Blue module hub genes were mainly enriched in signaling pathways such as regulation of cell-cell adhesion, axon guidance, neuron projection guidance, stem cell differentiation, and negative chemotaxis (**Figure 3I**). The hub genes of the Red module were mainly enriched in the negative regulation of inclusion body assembly, positive regulation of tumor necrosis factor-mediated signaling pathway, regulation of nucleotide-binding oligomerization domain containing signaling pathway, nucleotide-binding oligomerization domain containing 2 signaling pathway, regulation of inclusion body assembly and other signaling pathways (**Figure 3J**).

### The Core Regulatory Module of Natural Ovarian Ageing

**Figure 4:**
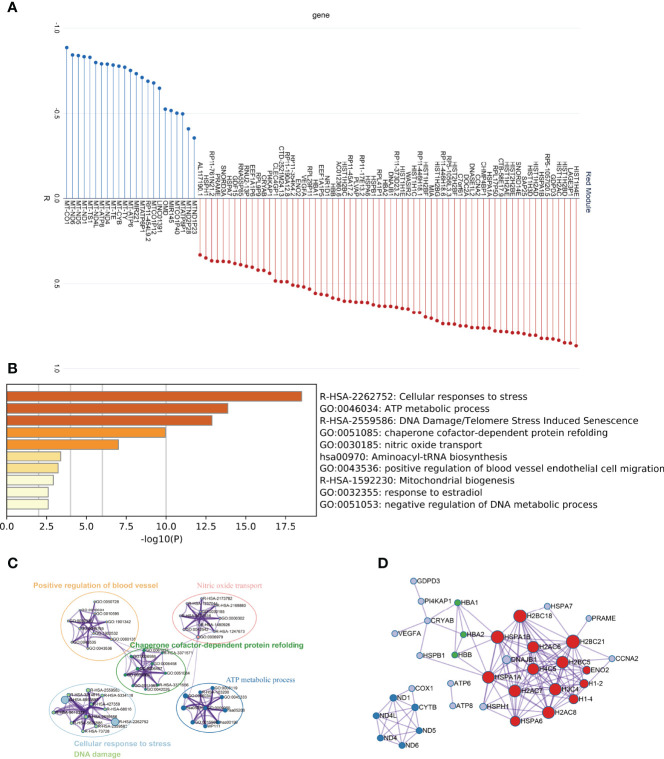
In-depth analysis of the Red module genes. **(A)** Correlation of Red module genes with the first principal component **(B, C)** Results of functional enrichment analysis. **(D)** PPI network and coregulatory modules.

As the red module is significantly and positively correlated with ovarian ageing, we carried out an in-depth analysis of the genes in the red module. Firstly, we found that the red module contains a large number of mitochondrial genes (**Figure 4A**). Metascape was used to perform functional enrichment analysis of the genes in the red module. The red module genes were mainly enriched in the Cellular response to stress, ATP metabolic process, DNA Damage/Telomere Stress induced Senescence, chaperone cofactor-dependent protein refolding, nitric oxide transport signaling pathways (**Figures 4B, C**). These signaling pathways have clear relevance to ageing. In addition, the red module of the gene constructed the PPI network, and the MCODE (26) algorithm was used to find the three pivotal modules of the PPI regulatory network (**Figure 4D**). The red hub module is mainly involved in Cellular responses to stress, DNA Damage/Telomere Stress Induced Senescence, and Cellular responses to stimuli signaling pathways. The blue hub module is mainly mitochondria-related genes (MT-ND1, MT-ND4L, MT-CYTB, MT-ND5, MT-ND6, MT-ND4), mainly involved in the aerobic electron transport chain, mitochondrial ATP synthesis coupled electron transport, ATP synthesis coupled electron transport signaling pathways. The green hub module (HBB, HBA1, HBA2) was mainly involved in the nitric oxide transport, Erythrocytes take up oxygen and release carbon dioxide, Scavenging of heme from plasma signaling pathway (**Table 1**).

**Table 1:** Functional enrichment of the core regulatory module of the protein-protein interaction network.

Network	Annotation
**MCODE_RED**	**R-HSA-2262752|Cellular responses to stress;R-HSA-2559586|DNA Damage/Telomere Stress Induced Senescence;R-HSA-8953897|Cellular responses to stimuli.**
**MCODE_GREEN**	**GO:0019646|aerobic electron transport chain;GO:0042775|mitochondrial ATP synthesis coupled electron transport;GO:0042773|ATP synthesis coupled electron transport.**
**MCODE_BLUE**	**GO:0030185|nitric oxide transport;R-HSA-1247673|Erythrocytes take up oxygen and release carbon dioxide;R-HSA-2168880|Scavenging of heme from plasma.**

In **Figure 4D**, MCODE_RED refers to the signal pathway enriched in the core genes of the PPI regulatory network in the red module; MCODE_GREEN refers to the signal pathway enriched in the core genes of the PPI regulatory network in the green module; MCODE_BLUE refers to the signal enriched in the core genes of the PPI regulatory network in the blue module communication.

### Aging of the Ovary in Fertile Women Is Associated With Activation of Inflammation-Related Signalling Pathways

**Figure 5:**
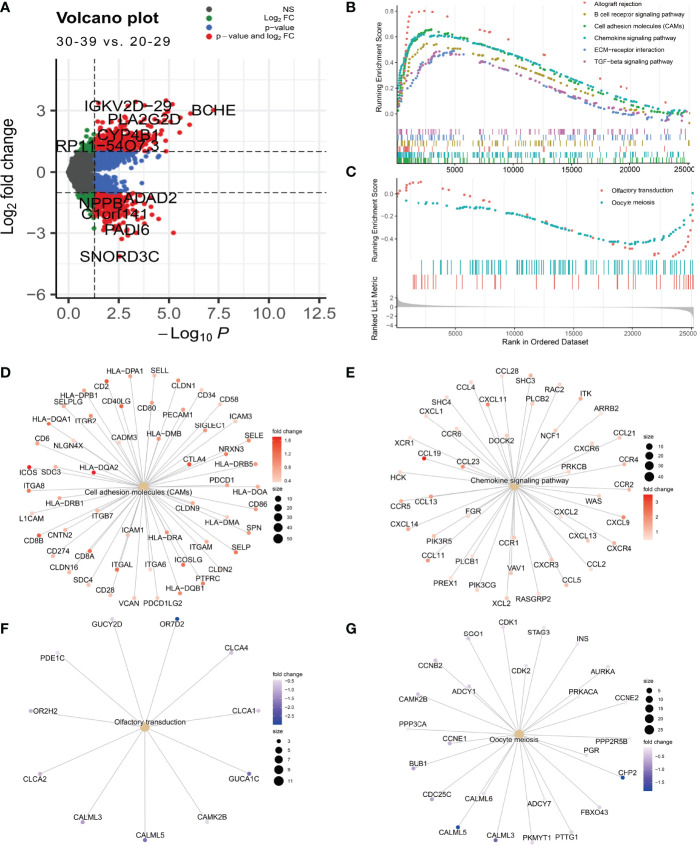
Differential expression analysis of the 0-29 age group and the 30-39 age group. **(A)** Differential expression volcano plot **(B)** Signaling pathways enriched in the 30-39 age group **(C)** Signaling pathways enriched in the 20-29 age group. **(D)** Cell adhesion moleculars (CAMs) coregulated genes. **(E)** Chemokine signaling pathway coregulated gene. **(F)** Olfactory transduction coregulator gene **(G)** Oocyte meiosis coregulator gene.

Fertile women are usually defined as those aged 20-39 years (27). Since women experience a significant decline in ovarian function after approximately 32 years of age, exploring the mechanisms of natural ovarian ageing in women aged 20-29 to 30-39 years is of great importance for reproductive health (3). We performed differential expression analysis on the expression profile data of ovarian tissues from the 20-29 and 30-39 groups. The results showed that the genes IGKV2D-29, BCHE, PLA2G2D, CYP4B1,and RP11-54O7.3 were highly expressed in ovarian tissues of 30-39-year-old women while SNORD3C, PADI6, C1orf141, NPPB, ADAD2 were lowly expressed (**Figure 5A**). The GSEA results showed that Allograft rejection, B cell receptor signaling pathway, Cell adhesion molecules (CAMs), Chemokine signaling pathway, ECM-receptor interaction, TGF-beta signaling pathway were significantly enriched in ovarian tissues of women aged 30-39 years (**Figure 5B**), while the signaling pathways of Olfactory transduction, Oocyte meiosis were mainly enriched in women aged 20-29 years (**Figure 5C**). This result demonstrates at the expression profiling level that women aged 30-39 years have a higher degree of ovarian inflammation and a reduced capacity for oocyte proliferation compared to women aged 20-29 years. In addition, we have demonstrated differential genes in the Celladhesion molecular, Chemokine signaling pathway, Olfactory transduction, and Oocyte meiosis signaling pathway (**Figures 5D–G**).

### Analysis of the Master Regulators of Aging in the Ovaries of Fertile Women

**Figure 6:**
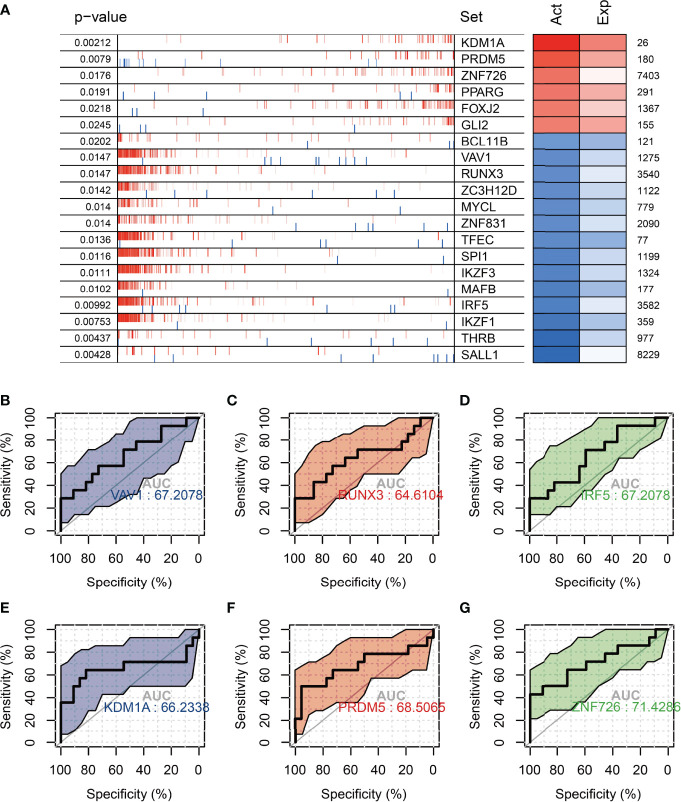
Analysis of master regulators in the 20-29 age group vs. the 30-39 age group. **(A)** Top 20 transcription factors with differential protein activity enrichment **(B–D)** Significantly activated transcription factors in the 30-39 age group for the diagnosis of ovarian senescence in the reproductive years (VAV1, RUNX3, IRF5). **(E–G)** Significantly activated transcription factors in the 20-29 age group for the diagnosis of ovarian senescence in the reproductive age group (KDM1A, PRDM5, ZNF726). Act: TF activation; Exp: TF expression.

In order to find the main regulatory factors that cause ovarian ageing in women and thus provide insight into the main causes of ovarian ageing in fertile women, we assessed changes in transcription factor activity through ovarian expression profiles in groups 20-29 and 30-39 using msviper. The results showed that transcription factors such as KDM1A, PRDM5, ZNF726, PPARG, FOXJ2, and GLI2 were significantly activated in the 20-29 group, while VAV1, RUNX3, ZC3H12D, MYCL, and IRF5 were significantly activated in the 30-39 group (**Figure 6A**). The activity and gene expression levels of these transcription factors were consistent with the results. Metascape was used to perform functional enrichment analysis of groups 30-39 transcription factors, and the results suggest that all of these transcription factors are regulatory hubs that promote inflammatory activation (**Table 2**). In addition, we constructed logistic regression models based on the transcription factor activity levels (calculated by viper) of VAV1, RUNX3, IRF5, KDM1A, PRDM5, and ZNF726, respectively, to predict age grouping (20-29 vs. 30-39, AUC=0.71, **Figures 6B–G**), whereas differentially expressed genes were poorly predicted (**Figures S2A–C**). This result suggests that the activity of these transcription factors could be an important indicator of ovarian ageing in fertile women.

**Table 2:** Functional enrichment results for transcription factors activated in the ovaries of women aged 30-39 years.

Description	Hits	Log(q-value)
**Hematopoietic stem cell differentiation**	**IRF5|SPI1|THRB|VAV1|IKZF1**	**-5.833782683**
**Lymphocyte activation**	**SPI1|VAV1|MAFB|IKZF1|IKZF3|BCL11B**	**-3.756866175**
**Leukocyte activation**	**SPI1|VAV1|MAFB|IKZF1|IKZF3|BCL11B**	**-3.37560533**
**Lymphocyte differentiation**	**SPI1|VAV1|MAFB|IKZF1|BCL11B**	**-3.37560533**
**Hemopoiesis**	**RUNX3|SPI1|VAV1|MAFB|IKZF1|BCL11B**	**-3.250822462**
**Mononuclear cell differentiation**	**SPI1|VAV1|MAFB|IKZF1|BCL11B**	**-3.250822462**
**Cell activation**	**SPI1|VAV1|MAFB|IKZF1|IKZF3|BCL11B**	**-3.184874215**
**Hematopoietic or lymphoid organ Development**	**RUNX3|SPI1|VAV1|MAFB|IKZF1|BCL11B**	**-3.184874215**
**Immune system development**	**RUNX3|SPI1|VAV1|MAFB|IKZF1|BCL11B**	**-3.08217932**

## Discussion

The natural ageing of the ovaries is a major threat to reproductive health in women (28). It has received increasing attention as the concept of delayed fertility has become popular (29). Recent studies on ovarian mechanisms of ageing have focused on DNA damage and mitochondrial dysfunction, telomere shortening, protein metabolism, and alterations in the stromal microenvironment (30, 31). However, the main subjects of these studies were mice, not humans. Therefore, these explanations of ovarian ageing mechanisms do not really reflect the biological mechanisms of natural ageing of the human ovary, especially the natural ageing pattern of the ovary in women of reproductive age.

First, we scored the degree of infiltration of 64 cell types in ovarian tissue samples from the 20-79 age group by XCELL. By comparing the 64 cell infiltration scores of ovaries from six age groups, 20-29, 30-39, 40-49, 50-59, 60-69, and 70-79, it was found that Endothelial cells, aDC cells, and Macrophages showed a significant increase in the age group 30-39 and then decreased with increasing age. In contrast, CD4+ Tcm, B cells steadily increase after the age of 50-59 years. It suggests that the mechanisms of ovarian ageing are quite different in the 20-49 age group and the 50-79 age group. In the 30-49 age group, the ovaries have significantly higher levels of inflammatory cells, while thereafter, the level of inflammation gradually decreases. Although elevated levels of inflammation have been demonstrated in mouse models (15), reductions in later inflammation levels have rarely been reported. Next, we used WGCNA to analyze the main regulatory gene modules in the natural ageing process of the ovary. We obtained six co-expression modules, of which the RED module showed a significant correlation with senescence. We found that mitochondrial genes were negatively associated with the Red module, suggesting that high expression of mitochondria-related genes is negatively associated with ageing. Mitochondrial damage is one of the major factors causing ovarian senescence. Mitochondrial damage is one of the main factors causing ageing. This contradiction is due to the fact that the ovarian samples included in this study included women in the age range 20-79 and that the ovarian ageing characteristics of women after the age of 50 show a very different pattern to that of fertile women. Comparing the 20-29 and 30-39 age groups reveals no significant differences in mitochondria-related genes between these two age groups (**Figure S1**). Thus, mitochondrial damage in the human ovary may not be a major cause of ovarian ageing during the reproductive years. We found that the Red module showed a significant positive correlation with age. We constructed a PPI network using 89 genes from the Red module and identified three of the major regulatory modules by using the MCODE algorithm. The red hub module consists of H1-2, H2AC6, HSPA6, H2AC8, H2BC5, H2BC21, H1-4, H3C4, H2BC18, H4C5, ENO2, H2AC7, HSPA1B, HSPA1A is mainly involved in the Cellular response to stress; DNA Damage/Telomere Stress Induced Senescence, Cellular responses to stimuli signaling pathways. The role of these signaling pathways in ageing has been extensively validated (31), however, the role of these genes in ovarian ageing still needs further exploration at present. The blue hub blocks of ND1, ND4L, ND4, ND6, ND5, and CYTB are mainly involved in the aerobic electron transport chain, mitochondrial ATP synthesis coupled electron transport, ATP synthesis coupled electron transport signaling pathway. However, from **Figure 4A**, we found that the red hub module and the blue hub module have opposite correlations with the first principal component of the red co-expression module. It suggests that age-regulated senescence is positively correlated with age for the red pivotal module (histone genes) and negatively correlated with age for the blue pivotal module (mitochondrial genes) throughout the aging process. These findings suggest that the decline in telomerase function and mitochondrial function are the main influencing factors in the ageing process between 20 and 79 years, however, the ageing of the ovaries in women of childbearing age (20-39) is not caused by these factors.

In addition, we found that in the 40-69 age group, the Endothelial cell score of the ovaries began to decrease. This condition may be a state of menopause due to ovarian atrophy. There are no relevant studies examining the relationship between ovarian inflammation and ovarian atrophy in women during menopause. Our study found a significant increase in B cells and CD4+ T cells during menopause. This result may suggest that ovarian atrophy during menopause may be associated with immune infiltration of B cells and CD4+ T cells.

The ovarian health status of women of reproductive age is one of the most important questions in the study of natural ovarian ageing. Therefore, in this study, we have conducted a further in-depth study of ovarian expression profiles in the 20-29 age group and 30-39 age group. We found that inflammation-related signaling pathways were significantly activated, and oocyte meiotic signaling pathways were significantly inhibited in women in the 30-39 age group. In addition, inflammation-related transcription factors (RUNX3, SPI1, IRF5, VAV1, BCL11B, etc.) were significantly activated in the 30-39 age group. In the previous discussion, we have already mentioned that the immune-related cells of ovarian cells are at their highest in the 30-39 age group. Therefore, we believe that the main cause of ovarian ageing in women aged 30-39 years is the activation of inflammation-related signaling pathways. We also found that classifiers based on transcription factor activity constructs (VAV1, RUNX3, IRF5) could distinguish between ovaries in the 20-29 and 30-39 age groups and were more effective than classifiers based on gene expression constructs. These results demonstrate that elevated levels of inflammation are the most important threat to the reproductive health of women aged 30-39 years. Mesenchymal stem cells have shown promising results in a variety of inflammation-induced diseases (32), and therefore the use of MSCs for ovarian maintenance in women aged 30-39 years may be a promising application (33).

Taken together, we identified the main cellular features and molecular mechanisms of ageing in the human ovary in the 20-79 age range. 20-49 and 50-79 year old women showed distinct patterns of ovarian ageing. In addition, we found that the main causes of ovarian senescence in women aged 20-39 were an increase in the proportion of immune cells and activation of inflammation-related signaling pathways. The main limitation of this study is that all conclusions are based on transcriptomic data from human ovaries and lack validation from relevant experiments. Nevertheless, it provides a research basis for exploring the cellular and molecular mechanisms of ovarian ageing in women and provides a theoretical basis for female reproductive health care.

## Data Availability Statement

The original contributions presented in the study are included in the article/**Supplementary Material**. Further inquiries can be directed to the corresponding author.

## Author Contributions

LC: Methodology, Software, Validation, Formal analysis, Investigation, Writing-Original Draft, Data Curation, Software, Validation. Writing- Review & Editing, Formal analysis. BD: Methodology. Software, Validation. Writing- Review & Editing, Formal analysis. LW: Supervision, Writing- Review & Editing, Formal analysis, Investigation, Methodology. JQ: Conceptualization, Project administration, Visualization, Supervision, Methodology. QL: Formal analysis, Investigation. ZY: Submission, Modification. JY: Conceptualization, Project administration, Visualization, Supervision, Methodology.

## Conflict of Interest

The authors declare that the research was conducted in the absence of any commercial or financial relationships that could be construed as a potential conflict of interest.

## Publisher’s Note

All claims expressed in this article are solely those of the authors and do not necessarily represent those of their affiliated organizations, or those of the publisher, the editors and the reviewers. Any product that may be evaluated in this article, or claim that may be made by its manufacturer, is not guaranteed or endorsed by the publisher.

## Supplementary Material

Supplementary Figure 1 No significant differences in mitochondria-related genes between the 20-39 and 30-39 age groups.

Supplementary Figure 2 Differentially expressed genes for diagnosis of ovarian senescence in women of reproductive age.
