# Supplementary figures and images for: Transcriptome Analysis Reveals the Mechanism of Natural Ovarian Ageing
Source: Front Endocrinol (Lausanne). 2022 Jul 14;13:918212. doi: 10.3389/fendo.2022.918212 (PMC9329525; doi:10.3389/fendo.2022.918212)

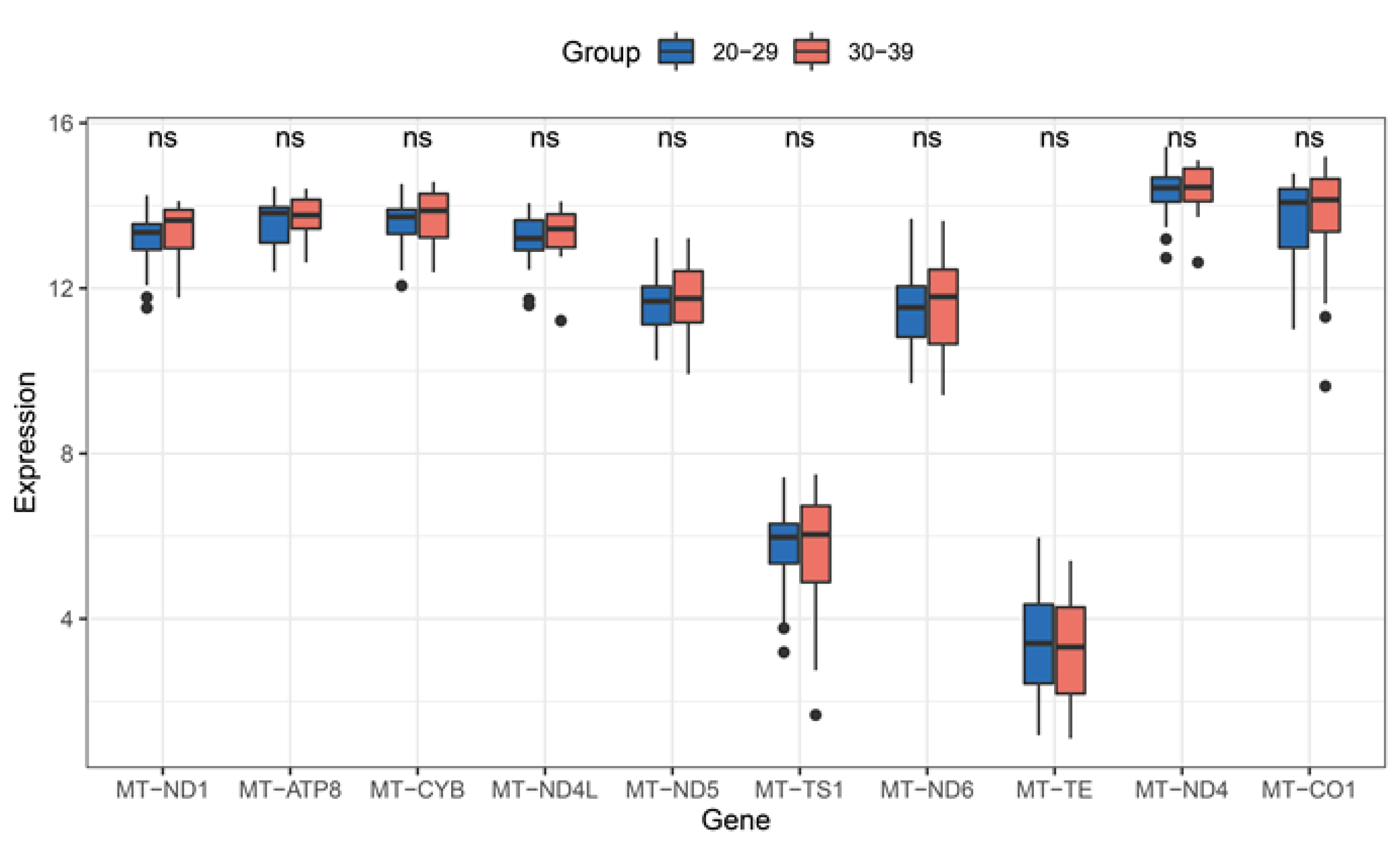

Supplement: Supplementary Figure 1 — No significant differences in mitochondria-related genes between the 20-39 and 30-39 age groups. [file Image_1.jpeg]

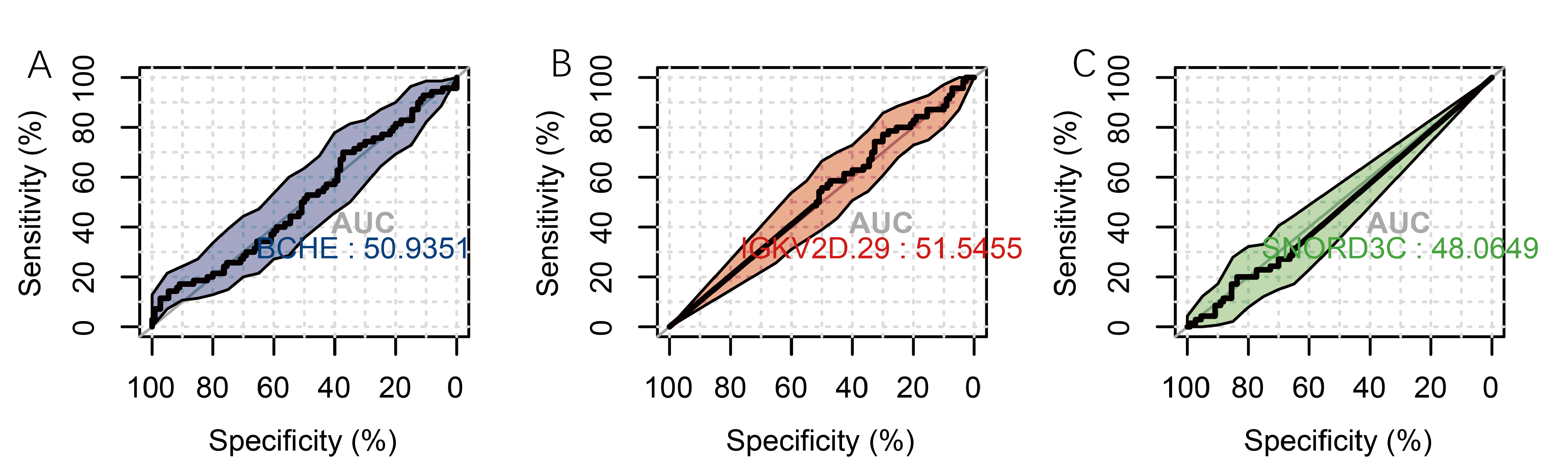

Supplement: Supplementary Figure 2 — Differentially expressed genes for diagnosis of ovarian senescence in women of reproductive age. [file Image_2.jpeg]
